# Regional differences between people who inject drugs in an HIV prevention trial integrating treatment and prevention (HPTN 074): a baseline analysis

**DOI:** 10.1002/jia2.25195

**Published:** 2018-10-22

**Authors:** Kathryn E Lancaster, Irving F Hoffman, Brett Hanscom, Tran Viet Ha, Kostyantyn Dumchev, Hepa Susami, Scott Rose, Vivian F Go, Sarah A Reifeis, Katie R Mollan, Michael G Hudgens, Estelle M Piwowar‐Manning, Paul Richardson, Sergii Dvoriak, Zubairi Djoerban, Tetiana Kiriazova, Oleksandr Zeziulin, Samsuridjal Djauzi, Chu Viet Ahn, Carl Latkin, David Metzger, David N Burns, Jeremy Sugarman, Steffanie A Strathdee, Susan H Eshleman, William Clarke, Deborah Donnell, Lynda Emel, Lisa E Sunner, Laura McKinstry, Nirupama Sista, Erica L Hamilton, Jonathan P Lucas, Bui D Duong, Nguyen Van Vuong, Riza Sarasvita, William C Miller, the HPTN 074 Study Team

**Affiliations:** ^1^ Division of Epidemiology College of Public Health The Ohio State University Columbus OH USA; ^2^ Division of Infectious Diseases School of Medicine The University of North Carolina at Chapel Hill Chapel Hill NC USA; ^3^ SCHARP‐FHCRC Seattle WA USA; ^4^ Department of Health Behavior Gilings School of Global Public Health The University of North Carolina at Chapel Hill Chapel Hill NC USA; ^5^ Ukrainian Institute on Public Health Policy Kyiv Ukraine; ^6^ University of Indonesia/Cipto Mangunkusumo Hospital Jakarta Indonesia; ^7^ FHI 360 Durham NC USA; ^8^ Department of Biostatistics Gilings School of Global Public Health The University of North Carolina at Chapel Hill Chapel Hill NC USA; ^9^ Center for AIDS Research (CFAR) School of Medicine The University of North Carolina at Chapel Hill Chapel Hill NC USA; ^10^ School of Medicine Johns Hopkins University Baltimore MD USA; ^11^ Academy of Labor, Social Relations and Tourism Kyiv Ukraine; ^12^ UNC Project Hanoi Vietnam; ^13^ Department of Health, Behavior, and Society Johns Hopkins University Baltimore MD USA; ^14^ Perelman School of Medicine University of Pennsylvania Philadelphia PA USA; ^15^ Division of AIDS National Institute of Allergy and Infectious Diseases U.S. National Institutes of Health Bethesda MD USA; ^16^ Department of Medicine Berman Institute of Bioethics Johns Hopkins University Baltimore MD USA; ^17^ Department of Medicine School of Medicine University of California San Diego San Diego CA USA; ^18^ Vietnam Authority of HIV/AIDS Control ‐ Ministry of Health Hanoi Vietnam; ^19^ Pho Yen Health District Center Pho Yen Vietnam; ^20^ National Narcotic Board Jakarta Indonesia

**Keywords:** injection drug use, PWID, HIV, substance use treatment, ART, treatment as prevention

## Abstract

**Introduction:**

People who inject drugs (PWID) experience high HIV incidence and face significant barriers to engagement in HIV care and substance use treatment. Strategies for HIV treatment as prevention and substance use treatment present unique challenges in PWID that may vary regionally. Understanding differences in the risk structure for HIV transmission and disease progression among PWID is essential in developing and effectively targeting intervention strategies of HIV treatment as prevention.

**Methods:**

We present a baseline analysis of HIV Prevention Trials Network (HPTN) 074, a two‐arm, randomized controlled trial among PWID in Indonesia (n = 258), Ukraine (n = 457) and Vietnam (n = 439). HPTN 074 was designed to determine the feasibility, barriers and uptake of an integrated intervention combining health systems navigation and psychosocial counselling for the early engagement of antiretroviral therapy (ART) and substance use treatment for PWID living with HIV. Discordant PWID networks were enrolled, consisting of an HIV‐positive index and their HIV‐negative network injection partner(s). Among the enrolled cohort of 1154 participants (502 index participants and 652 network partners), we examine regional differences in the baseline risk structure, including sociodemographics, HIV and substance use treatment history, and injection and sexual risk behaviours.

**Results:**

The majority of participants were male (87%), with 82% of the enrolled females coming from Ukraine. The overall mean age was 34 (IQR: 30, 38). Most commonly injected substances included illegally manufactured methadone in Ukraine (84.2%), and heroin in Indonesia (81.8%) and Vietnam (99.5%). Injection network sizes varied by region: median number of people with whom participants self‐reported injecting drugs was 3 (IQR: 2, 5) in Indonesia, 5 (IQR: 3, 10) in Ukraine and 3 (IQR: 2, 4) in Vietnam. Hazardous alcohol use, assessed using the Alcohol Use Disorders Identification Test – Alcohol Consumption Questions (AUDIT‐C), was prominent in Ukraine (54.7%) and Vietnam (26.4%). Reported sexual risk behaviours in the past month, including having two or more sex partners and giving/receiving money or drugs in exchange for sex, were uncommon among all participants and regions.

**Conclusions:**

While regional differences in risk structure exist, PWID particularly in Ukraine need immediate attention for risk reduction strategies. Substantial regional differences in risk structure will require flexible, tailored treatment as prevention interventions for distinct PWID populations.

## 1 Introduction

HIV epidemics in eastern Europe, central Asia and many parts of South East Asia are concentrated among people who inject drugs (PWID) 1. Serial use and sharing of drug preparation and injection equipment create heightened risks for acquiring and transmitting HIV 2, 3. The persistently high incidence of HIV infection among PWID in many locations with concentrated epidemics necessitates aggressive efforts to prevent HIV transmission 1.

Given the ethical complexity of mitigating stigma and legal risks and providing effective harm reduction services, PWID have been largely excluded from HIV prevention trials 4, 5, 6. Although HIV Prevention Trials Network (HPTN) 052 demonstrated the benefit of early antiretroviral therapy (ART) among people living with HIV to prevent sexual HIV transmission 7, the trial excluded active PWID. Consequently, the concept of treatment as prevention, in which individuals are treated with ART to prevent transmission of HIV to others, has not been validated in PWID living with HIV. Parenteral exposure to HIV during injection drug use typically has a higher infectious dose, leading to a higher transmission probability 8. Thus, the effectiveness of ART to reduce HIV transmission may be lower among PWID than the 96% reduction in HIV found among HIV discordant sexual partnerships in HPTN 052 7.

Testing HIV treatment as prevention strategies among PWID presents unique challenges. HIV transmission in PWID occurs in the context of risk networks, typically with the involvement of multiple injection partners, varied injection practices and sexual risk behaviours 9, 10. Injection behaviours among PWID after initiating ART may change due to alterations in risk behaviours or increased attention to health concerns leading to a reduction in injection related risks or an increased use of sterile injection equipment 11, 12, 13. Continued substance use may also lead to poor ART adherence and retention, treatment failure and transmission of resistant strains 14, 15, 16. To maximize the potential for treatment success, novel approaches for offering ART in conjunction with substance use treatment modalities to PWID must be explored, particularly across multiple regions 1, 17, 18, 19.

PWID in need of HIV care across varying regions likely experience different risk structures for HIV transmission and disease progression. In Indonesia, Ukraine and Vietnam, the HIV epidemic is primarily concentrated among PWID 20, 21, 22, 23. Yet, differences in sociodemographics and injection substances and practices that increase risk may highlight the need for region‐specific strategies to prevent HIV transmission 3, 24. An enhanced understanding of risk structure among PWID within these countries will directly inform treatment as prevention interventions that are flexible enough to address potential regional differences. HPTN 074 was designed to determine the feasibility, barriers and uptake of a multisite, integrated intervention combining supported referrals and brief psychosocial counselling for the early engagement of ART and substance use treatment for PWID living with HIV. HPTN 074 enrolled HIV discordant PWID networks of HIV‐positive PWID with unsuppressed viraemia and their HIV‐negative PWID injection partners in three geographically and culturally distinct regions: Indonesia, Ukraine and Vietnam.

Here, we examine regional differences in the baseline risk structure, including sociodemographics, HIV and substance use treatment history, and injection and sexual risk behaviours, among the HPTN 074 study cohort.

## 2 Methods

### 2.1 Study settings

This study was conducted among PWID in three distinct locations with documented HIV epidemics among PWID: Jakarta, Indonesia; Kyiv, Ukraine; and Thai Nguyen, Vietnam. These study sites were chosen based on HIV prevalence and incidence among PWID. In Jakarta, the estimated prevalence among PWID is 54%. In Kyiv, HIV prevalence among the estimated 31,300 PWID was 20% in 2013; incidence observed in a prospective study was 4.5 (95% CI: 2.3, 7.9) per 100 person‐years (PY) 20, 21, 22. Among PWID in Thai Nguyen 2005 to 2007 trial, the calculated HIV prevalence was 35% and HIV seroconversion incidence rate was 5.2 (95% CI: 3.5, 7.6) per 100 PY 23.

### 2.2 Study population

The study population included PWID networks with two participants’ types: index participants living with HIV with unsuppressed viraemia (≥1000 copies/mL) and their HIV‐negative network injection partners 25. The inclusion criteria for both types of participants included: male or female gender, age between 18 and 60 years (the upper age limit was increased from 45 years to 60 years in September 2015 26); active injection drug use (defined initially as self‐report of (i) injecting drugs approximately two or more times per week for the past three months and (ii) ability to identify the anatomical location of the most recent injection site that was confirmed by site research staff; updated in September 2015 to (i) injecting 12 times or more in the past three months and at least six times in the past month and (ii) a PWID in the opinion of site research staff); having no plans to move outside the study area for at least one year after study enrolment; and ability to provide written informed consent. Inclusion criteria specific for index participants also included: HIV infection based on local standard of care testing; viral load ≥1000 copies/mL and CD4 cell count >50 cells/μL at screening; willingness and ability to identify, recruit and enrol at least one HIV‐negative network injection partner who was eligible for study participation; and willingness to participate in intervention activities including regular phone contact 25.

### 2.3 Parent study design

This multisite, two‐arm, randomized study was designed to: (1) determine the feasibility of a future randomized controlled trial by estimating HIV incidence among network partners and assessing the potential for enrolment and retention of PWID living with HIV and their HIV‐network partners; and (2) assess the feasibility, barriers and uptake of the integrated interventions 25.

Index participants in the intervention arm received a standard harm reduction package and an integrated intervention that included: (1) systems navigators to facilitate engagement, retention and adherence in substance use treatment and HIV care; (2) psychosocial counselling to facilitate substance use treatment and HIV care and medication adherence; and (3) referral for ART at any CD4 count. Index participants in the standard of care arm received the World Health Organization (WHO) package of care for PWID, including HIV testing and counselling and referrals for ART, diagnosis and treatment of sexually transmitted infections, hepatitis B and C virus, and tuberculosis, as appropriate. All network partners received a standardized harm reduction package with referral for syringe service programmes and substance use treatment, consistent with national guidelines.

### 2.4 Recruitment procedures for index participants

Index participants were recruited using a variety of methods, including referral from HIV‐testing sites, community outreach and injection‐network referrals. Trained outreach workers who were knowledgeable about community dynamics, including geographic areas, settings and organizations frequented by PWID, were selected from the community and harm reduction programmes. Outreach workers were trained on basic methods of rapid assessment procedures to target areas of high drug use. These workers disseminated information about the study, provided oral and written descriptions of the study to prospective participants and encouraged index participants to participate in screening activities at the local study site. Potential index participants were asked to share the information to other PWID in their networks.

### 2.5 Identification of network partners

After confirmation of positive HIV status via local standard of care testing, index participants were asked to identify members of their injection network with whom they engage in HIV‐related injection exposures, such as sharing injection equipment. Index participants were provided with referral identification cards, which did not contain identifying information, and encouraged to accompany network partners to the study site. Index participants were asked to provide descriptions, such as name, age and gender, of members of their injection network with whom they may provide referral identification cards. Up to five HIV‐negative network partners per index participant were allowed to enrol. Network partners had to match the description provided by the index to participate in the screening process and were then asked to provide study informed consent for enrolment and to be tested for HIV infection. Index participants received compensation for successful enrolment of network partners. Network partners were compensated for their time and participation. The amount and form of compensation was approved by the local institutional review boards (IRB) and varied by site.

### 2.6 Cohort enrolment

**Figure 1:**
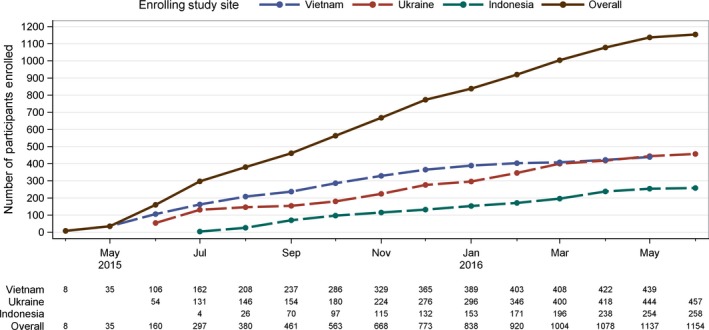
Cumulative enrolment by site in HPTN 074 (N = 1154)

Enrolment began in April 2015 in Vietnam, June 2015 in Ukraine and July 2015 in Indonesia (Figure 1). The study enrolled 504 network units (504 index participants, 656 baseline network partners). Of the 504 enrolled index participants, one had a viral load <1000 copies/mL at screening and was excluded from analyses. Of the 656 enrolled network partners, four participants were excluded; the network partner of the excluded index and three other network partners who were found to be HIV positive at a screening based on testing performed retrospectively at the HPTN LC. One of the excluded network partners was the sole partner of an index and that index participant was removed from the analyses. The final baseline analysis sample included 1154 participants (502 network units: 502 index participants and 652 network partners).

### 2.7 Data collection

At screening, index participants provided blood samples for HIV viral load testing and CD4 cell counts. After enrolment consent was obtained, index participants and network partners completed face‐to‐face interviews with study staff who had extensive experience with non‐judgemental interviewing techniques.

### 2.8 Measures

All participants provided information on sociodemographics characteristics: sex, age, marital status, highest level of education completed and employment. Participants self‐reported HIV testing and treatment history, including ART use (currently on ART, previously on ART or ART naïve) and date of their HIV diagnosis. Years since HIV diagnosis was calculated based on the difference of dates between HIV diagnosis and survey completion. Baseline substance use measures were collected for the prior three months and included: alcohol use, non‐injection and injection drug use, and number of people used drug with. The measure used to define whether participants displayed hazardous alcohol use was the Alcohol Use Disorders Identification Test – Alcohol Consumption Questions (AUDIT‐C) score 27. Males with AUDIT‐C scores ≥4 and females with scores ≥3 were classified as displaying hazardous alcohol use behaviours 28. Injection practices were collected for last injection, which included shared rinse water, shared cooker/container, shared filter cotton, used a new needle, cleaned needle before injection, used pre‐filled syringe and injected drugs that were frontloaded or backloaded into the syringe. Participants reported whether they had ever participated methadone maintenance or any other medication‐assisted treatment (MAT) programme. Sexual behaviour measures, including number of reported sexual partners and number of times giving and receiving sex for money, were collected for the prior month. Additional testing was performed at the HPTN Laboratory Center (LC), Johns Hopkins University, Baltimore, MD, using FDA‐cleared assays; baseline testing included confirmation of HIV status for index and network partners and viral load testing.

### 2.9 Statistical analysis

Frequency distributions and descriptive statistics were used to summarize enrolment, sociodemographic characteristics, HIV testing and treatment history, self‐reported substance use and treatment history, and injection and sexual behaviours of the enrolled study cohort at baseline. Pearson's chi‐square test was used to evaluate differences in baseline categorical variables by region, with Fisher's exact test used when any expected cell counts were less than 5. Continuous measures were compared using one‐way ANOVA. Analyses were conducted in Linux SAS version 9.4(SAS/STAT 14.2, Cary, NC).

### 2.10 Ethics approval

The study protocol, which is available at clinicaltrials.gov (NCT02935296), was approved by at least one local IRB affiliated with each site: University of Indonesia, Ukrainian Institute on Public Health Policy, Thai Nguyen Center for Preventive Medicine, University of North Carolina‐Chapel Hill and Faculty of Medicine. All study participants provided written informed consent in their local languages, or English, if preferred.

## 3 Results

### 3.1 Sociodemographic characteristics of the enrolled cohort

The baseline cohort, including index and network partners, was predominantly male (87.3%), with most females enrolled at the Ukraine site (Table 1). The median age at enrolment was 34 years (IQR: 30, 38). Unlike Indonesia and Vietnam, most Ukrainian participants were married or living with a sexual partner (57.7%). Overall, 54.7% of participants in Indonesia and 41.8% in Ukraine completed college or technical college, compared to only 4.8% in Vietnam. Most Ukrainian participants were unemployed (65.0%), while most participants in Indonesia (73.6%) and Vietnam (77.5%) were employed either full‐ or part‐time.

**Table 1:** Baseline sociodemographic characteristics by site in HPTN 074

	Indonesian = 258		Ukrainen = 457		Vietnam n = 439		Total n = 1154		*p*‐value^a^
	n	(%)	n	(%)	n	(%)	n	(%)	
Sex									
Male	238	(92.2)	337	(73.7)	432	(98.4)	1007	(87.3)	<0.01
Female	20	(7.8)	120	(26.3)	7	(1.6)	147	(12.7)	
Age (years)									
18 to 19	5	(1.9)	0	(0.0)	1	(0.2)	6	(0.5)	<0.01
20 to 29	88	(34.1)	83	(18.2)	98	(22.3)	269	(23.3)	
30 to 39	137	(53.1)	287	(62.8)	225	(51.3)	649	(56.2)	
40 to 45	23	(8.9)	80	(17.5)	101	(23.0)	204	(17.7)	
>45	5	(1.9)	7	(1.5)	14	(3.2)	26	(2.3)	
Marital status									
Married	96	(37.2)	102	(22.3)	203	(46.2)	401	(34.7)	<0.01
Living with sexual partner but not married	7	(2.7)	162	(35.4)	2	(0.5)	171	(14.8)	
Separated, divorced, widowed	61	(23.6)	64	(14.0)	96	(21.9)	221	(19.1)	
Single	94	(36.4)	129	(28.2)	138	(31.4)	361	(31.3)	
Education									
No schooling or some primary school	14	(5.4)	0	(0.0)	36	(8.2)	50	(4.3)	<0.01
Completed primary school or some secondary school	32	(12.4)	35	(7.7)	279	(63.6)	346	(30.0)	
Completed secondary school or some technical training/college/university	71	(27.5)	231	(50.5)	103	(23.5)	405	(35.1)	
Completed technical training or college/university	141	(54.7)	191	(41.8)	21	(4.8)	353	(30.6)	
Employment^b^									
Employed full‐time	79	(30.6)	66	(14.4)	222	(50.6)	367	(31.8)	<0.01
Employed part‐time	111	(43.0)	89	(19.5)	118	(26.9)	318	(27.6)	
Unemployed but seeking work	57	(22.1)	214	(46.8)	64	(14.6)	335	(29.0)	
Unemployed – not seeking work	11	(4.3)	83	(18.2)	34	(7.7)	128	(11.1)	
Retired	0	(0.0)	4	(0.9)	1	(0.2)	5	(0.4)	

a Pearson's chi‐square test was used to test association between each row variable and site; Fisher's exact test was used when any of the expected cell counts were less than 5 or when 0 counts were present.

b Refused to answer, n = 1 (Ukraine).

### 3.2 HIV treatment history of index participants

Among HIV‐positive index participants, the median time since their self‐reported HIV diagnosis was longer in Ukraine (4.16 years, IQR: 1.87, 9.37; Table 2), compared to Indonesia (1.50 years, IQR: 0.02, 6.91) and Vietnam (0.09 years, IQR: 0.06, 0.45). Across all three sites, most index participants (~70% of in Indonesia and >80% in Ukraine and Vietnam) reported that they were ART naïve at enrolment. The median viral load of all index participants at screening was 4.56 log_10_ copies/mL (IQR: 3.99, 4.99), with a narrow range of medians among sites (4.53, 4.61). The median baseline CD4 count for index participants across all sites at screening was 293 cells/μL overall (IQR: 166, 463). The median baseline CD4 count was lowest in Indonesia (271 cells/μL; IQR: 147, 418) and highest in Vietnam (314 cells/μL; IQR: 187, 492).

**Table 2:** Baseline antiretroviral drug use, CD4 cell count and HIV viral load among index participants in HPTN 074 (n = 502)

	Indonesian = 121		Ukrainen = 187		Vietnam n = 194		Total n = 502		*p*‐value^a^
	N (%)		N (%)		N (%)		N (%)		
ART use^b^									
Currently on ART	23 (19.0)		3 (1.6)		28 (14.4)		54 (10.8)		<0.01
Previously on ART	14 (11.6)		28 (15.0)		4 (2.1)		46 (9.2)		
ART naïve	84 (69.4)		156 (83.4)		162 (83.5)		402 (80.1)		
	**Median**	**(IQR)**	**Median**	**(IQR)**	**Median**	**(IQR)**	**Median**	**(IQR)**	
Years since HIV diagnosis	1.50	(0.02,6.91)	4.16	(1.87,9.37)	0.09	(0.06,0.45)	1.41	(0.07,6.41)	<0.01
HIV‐1 viral load (log_10_ copies/mL)	4.53	(4.18, 4.94)	4.54	(3.83, 5.07)	4.61	(4.05, 4.96)	4.56	(3.99, 4.99)	0.99
CD4 cell count (cells/μL)	271	(147, 418)	310	(178, 465)	314	(187, 492)	293	(166, 463)	0.09

IQR: interquartile range; ART: antiretroviral therapy.

a Pearson's chi‐square test was performed for ART use and one‐way ANOVA was used for continuous measures.

b Based on self‐report.

### 3.3 Risk structure of enrolled cohort

Among all index participants and network partners at all three sites, recent substance use was common (Table 3). In addition to injection drug use, hazardous alcohol use was uncommon in Indonesia (9.3%), but prominent in Ukraine (54.7%) and Vietnam (26.4%). Marijuana use in the past three months was common among Indonesian (41.5%) and Ukrainian (64.8%) participants, but substantially less common in Vietnam (0.9%). Three‐quarters (75.6%) of Indonesian participants reported recent (prior three months) non‐injectable stimulant use, as compared to only 28.4% in Ukraine and 18.0% in Vietnam. The most commonly injected drugs were heroin (81.8%) and buprenorphine (37.6%) in Indonesia; illegally manufactured methadone (84.2%), home‐made opioids (75.7%) and amphetamines (35.7%) in Ukraine; and heroin (99.5%) in Vietnam.

**Table 3:** Baseline substance use behaviours by site in HPTN 074 (n = 1154)

	Indonesian = 258	Ukrainen = 457	Vietnamn = 439	Totaln = 1154	*p*‐value^a^
	n (%)	n (%)	n (%)	n (%)	
Hazardous alcohol use^b^	24 (9.3)	250 (54.7)	116 (26.4)	390 (33.8)	<0.01
Non‐injection drug use, past three months^c^					
Marijuana	107 (41.5)	296 (64.8)	4 (0.9)	407 (35.3)	<0.01
Stimulants (cocaine, methamphetamines)	195 (75.6)	130 (28.4)	79 (18.0)	404 (35.0)	
Opiates	30 (11.6)	21 (4.6)	55 (12.5)	106 (9.2)	
Benzodiazepine	136 (52.7)	2 (0.4)	0 (0.0)	138 (12.0)	
Methadone (illegally manufactured)	0 (0.0)	4 (0.9)	0 (0.0)	4 (0.3)	
Injection drug use, past three months^c^					
Amphetamines	2 (0.8)	163 (35.7)	1 (0.2)	166 (14.4)	<0.01
Heroin	211 (81.8)	40 (8.8)	437 (99.5)	688 (59.6)	
Opium	3 (1.2)	58 (12.7)	0 (0.0)	61 (5.3)	
Buprenorphine	97 (37.6)	65 (14.2)	0 (0.0)	162 (14.0)	
Methadone (illegally manufactured)	4 (1.6)	385 (84.2)	1 (0.2)	390 (33.8)	
Home‐made opioids	0 (0.0)	346 (75.7)	0 (0.0)	346 (30.0)	
Shared rinse water, last injection					
Yes	149 (57.8)	88 (19.3)	15 (3.4)	252 (21.8)	<0.01
No	109 (42.2)	369 (80.7)	424 (96.6)	902 (78.2)	
Shared cooker/container, last injection^d^					
Yes	149 (57.8)	301 (65.9)	116 (26.4)	566 (49.0)	<0.01
No	109 (42.2)	155 (33.9)	323 (73.6)	587 (50.9)	
Shared filter cotton, last injection^d^					
Yes	3 (1.2)	254 (55.6)	1 (0.2)	258 (22.4)	<0.01
No	255 (98.8)	202 (44.2)	437 (99.5)	894 (77.5)	
Used a new needle, last injection					
Yes	208 (80.6)	398 (87.1)	393 (89.5)	999 (86.6)	<0.01
No	50 (19.4)	59 (12.9)	46 (10.5)	155 (13.4)	
Cleaned needle before injection, last injection^d^					
Yes	106 (41.1)	52 (11.4)	71 (16.2)	229 (19.8)	<0.01
No	152 (58.9)	403 (88.2)	367 (83.6)	992 (86.0)	
Used a pre‐filled syringe, last injection					
Yes	140 (54.3)	90 (19.7)	26 (5.9)	256 (22.2)	<0.01
No	118 (45.7)	367 (80.3)	413 (94.1)	898 (77.8)	
Injected drugs that were frontloaded or backloaded into the syringe or needle, last injection^d^					
Yes	254 (98.4)	372 (81.4)	102 (23.2)	728 (63.1)	<0.01
No	4 (1.6)	84 (18.4)	337 (76.8)	425 (36.8)	
Number of people used drugs with, past three months					
0	0 (0.0)	0 (0.0)	0 (0.0)	0 (0.0)	<0.01
1	22 (8.5)	17 (3.7)	69 (15.7)	108 (9.4)	
2 to 4	170 (65.9)	166 (36.3)	321 (73.1)	657 (56.9)	
≥5	66 (25.6)	274 (60.0)	49 (11.2)	389 (33.7)	
Ever participated in methadone maintenance or any other medication‐assisted treatment programme					
Yes	138 (53.5)	214 (46.8)	99 (22.6)	451 (39.1)	<0.01
No	120 (46.5)	243 (53.2)	340 (77.4)	703 (60.9)	

a Pearson's chi‐square test was used to test association between each row variable and site; Fisher's exact test was used when any of the expected cell counts were less than 5 or when 0 counts were present.

b Hazardous alcohol use was determined as an Alcohol Use Disorders Identification Test – Alcohol Consumption Questions (AUDIT‐C) score of ≥4 among males and ≥3 among females.

Participants may report more than one substance type.

Missing data due to not knowing or refused to answer: shared cooker/container, n = 1; shared filter cotton, n = 2; cleaned needle before injection, n = 3; injected drugs that were frontloaded or backloaded into the syringe or needle, n = 1.

Injection risk behaviours were common among index participants and network partners across all three sites. In Indonesia, 57.8% reported sharing rinse water and sharing a cooker/container at last injection. Sharing of filter cotton at last injection was more common in Ukraine (55.6%) than in Indonesia (1.2%) and Vietnam (0.2%). Most participants at all three sites (80.6% in Indonesia, 87.1% in Ukraine and 89.5% in Vietnam) reported using a new needle at last injection.

Many of the participants in Ukraine (60.0%) reported using injection drugs with five or more different people in the prior three months. Participants in Indonesia and Vietnam reported using drugs with a median of three people (IQR: 2, 5 in Indonesia; IQR: 2, 4 in Vietnam). Participants in Ukraine reported using drugs with a median number of five people (IQR: 3, 10).

Almost half (46.5%) of participants in Indonesia, over half in Ukraine (53.2%) and over three‐quarters in Vietnam (77.4%) reported never having participated in methadone maintenance or any other MAT programme prior to enrolment.

Reported sexual risk behaviours were uncommon among all participants (Table 4). Nearly all male participants (91.5%) reported one or no female sexual partners in the past month. Similarly, 94.6% of female participants reported one or no male sexual partners in the past month. Overall, 3.1% of male participants and zero female participants reported giving money or drugs in exchange for sex, and 0.8% of male participants and 2.0% of female participants reported receiving money or drugs in exchange for sex in the past month.

**Table 4:** Baseline sexual risk behaviours in past month by site in HPTN 074 (N = 1154)

	Indonesia		Ukraine		Vietnam		Total	
	Male^a^ n = 238	Female n = 20	Male n = 337	Female n = 120	Male n = 432	Female n = 7	Male^a^ n = 1007	Female n = 147
	n (%)	n (%)	n (%)	n (%)	n (%)	n (%)	n (%)	n (%)
Number of different female sex partners								
0	95 (39.9)	20 (100.0)	60 (17.8)	117 (97.5)	215 (49.8)	7 (100.0)	370 (36.7)	144 (98.0)
1	130 (54.6)	0 (0.0)	234 (69.4)	3 (2.5)	187 (43.3)	0 (0.0)	551 (54.8)	3 (2.0)
≥2	12 (5.0)	0 (0.0)	43 (12.8)	0 (0.0)	30 (6.9)	0 (0.0)	85 (8.4)	0 (0.0)
Number of different male sex partners								
0	237 (99.6)	3 (15.0)	337 (100.0)	35 (29.2)	432 (100.0)	2 (28.6)	1006 (99.9)	40 (27.2)
1	0 (0.0)	13 (65.0)	0 (0.0)	81 (67.5)	0 (0.0)	5 (71.4)	0 (0.0)	99 (67.4)
≥2	0 (0.0)	4 (20.0)	0 (0.0)	4 (3.3)	0 (0.0)	0 (0.0)	0 (0.0)	8 (5.4)
Number of times giving sex partners money or drugs in exchange for sex								
0	224 (94.1)	20 (100.0)	330 (97.9)	120 (100.0)	422 (97.7)	7 (100.0)	976 (96.9)	147 (100.0)
1	2 (0.8)	0 (0.0)	2 (0.6)	0 (0.0)	4 (0.9)	0 (0.0)	8 (0.8)	0 (0.0)
≥2	12 (5.0)	0 (0.0)	5 (1.5)	0 (0.0)	6 (1.4)	0 (0.0)	23 (2.3)	0 (0.0)
Number of times receiving money or drugs in exchange for sex								
0	229 (96.2)	19 (95.0)	337 (100.0)	118 (98.4)	432 (100.0)	7 (100.0)	998 (99.1)	144 (98.0)
1	2 (0.8)	0 (0.0)	0 (0.0)	1 (0.8)	0 (0.0)	0 (0.0)	2 (0.2)	1 (0.7)
≥2	6 (2.5)	1 (5.0)	0 (0.0)	1 (0.8)	0 (0.0)	0 (0.0)	6 (0.6)	2 (1.3)

a Missing data due to not knowing or refused to answer, n = 1.

## 4 Discussion

To our knowledge, this study represents one of the largest multisite cohorts of PWID living with HIV and their HIV‐negative injection partners in an HIV prevention trial. Several significant regional differences in PWID risk structure exist across Indonesia, Ukraine and Vietnam. Notably, PWID in Ukraine may be uniquely vulnerable to a continued HIV epidemic given the proportion of female PWID, time since HIV diagnosis and need for HIV care for viral suppression, substance use variation and density of drug use networks. These findings have important implications for HIV and substance use treatment strategies among PWID worldwide.

Although the sociodemographic characteristics of participants were largely similar in each country, PWID in Vietnam reported lower education and Ukraine had a significantly larger proportion of female PWID. For PWID in Vietnam, low levels of education may serve as a barrier for HIV and substance use treatment 29. We believe the limited number of females in Indonesia and Vietnam accurately reflects the population of PWID in these countries based on culture and historic precedence 30, 31, 32. The gender distribution in Ukraine is similar to the distribution in Russia and the Baltic States 27, 33. Female PWID often face more stigma and discrimination than their male PWID, which can be an additional barrier for engaging in HIV and substance use treatment 34. Treatment as prevention interventions, as well as substance use treatment, should address vary education levels and integrate female tailored approaches, where appropriate.

Variations in HIV status awareness highlight unique regional needs for routine HIV testing and counselling. Index participants in Ukraine reported that they knew their HIV status for a median of over four years, compared to one and a half years in Indonesia and less than one year in Vietnam. This difference was assumably due to barriers in receiving routine HIV testing for PWID, especially in Vietnam 35, 36, 37. In Ukraine, the primary recruitment location was staffed by an organization that fostered long‐term relationships with PWID and provided routine HIV testing and counselling and needle exchange. Additional efforts should be made to leverage relationships and enhance engagement and adherence to HIV treatment across all regions.

The significant variability in both injectable and non‐injectable substance likely indicates availability and affordability of drugs at the time of enrolment. Heroin in Indonesia and Vietnam is typically cheap and available, given the proximity to the Golden Triangle where the borders of Thailand, Laos and Myanmar meet and opium trafficking is prominent 38. In contrast, in Ukraine, heroin is expensive and less available than other substances, such as home‐made opiates 39, 40. Over half of Ukrainian participants reported hazardous alcohol use, while two‐thirds reported using marijuana. Additionally, methadone, home‐made opioids and amphetamines were the predominant injected substances. The extensive use of varying types of substances, may delay engagement and hinder benefits of HIV and substance use treatment among PWID 41, 42, 43.

The density of injection drug networks in Ukraine depicts potential HIV transmission dynamics in need of appropriate prevention interventions. The size of the injection drug networks appears to be larger in Ukraine than in Indonesia or Vietnam. Injection network size likely reflects the social norms related to injection behaviour in each area 44. Large, dense networks are associated with injection practices that increase the risk for HIV transmission 45, 46, 47. The larger networks in Ukraine may arise because of the uncertainty of the drug sources and the culture of home‐made drug preparation 39, 40, 48. Further examination of contact patterns within these networks will identify target for HIV treatment as prevention interventions 49.

Within our study population, injection risk behaviours were more commonly reported than sexual risk behaviours across all three sites. However, this does not necessarily suggest injection as the primary mode of potential HIV transmission. Given recruitment and enrolment targeted PWID, participants may have felt comfortable reporting injection behaviours. Additionally, sexual risk behaviours are likely under‐reported due to the stigmatization, particularly among sexual relationships of the same sex or outside of marriage 50, 51, 52.

The high prevalence of injection risk behaviours across all sites emphasizes the importance of integrating effective harm reduction with innovative strategies to prevent ongoing transmission. Sharing injection equipment significantly increases the risk of HIV acquisition among PWID 53, 54, 55. Harm reduction approaches, such as needle and syringe exchange programmes and MAT, can significantly reduce the number of new HIV infections, even when coverage is high 56, 57, 58. Access and use of MAT appears to be less in Vietnam where almost four out of five PWID had never participated in a substance use programme, compared to approximately half in Indonesia and Ukraine. In all three countries, the number of available MAT clinics is increasing due to changes in health policy 59, 60, 61. Consequently, uptake of MAT services among the enrolled cohort may have increased throughout study follow‐up.

We characterized a cohort of HIV‐infected PWID and their HIV‐uninfected network partners in three distinct settings, providing insights on the risk structure of the study population. Enrolled PWID may not be generalizable to PWID who were not reached or consented within each region. Recruitment efforts may have varied, particularly in terms of access to PWID populations, despite the structured and coordinated recruitment strategies across each site. This may have resulted in differences in the risk structure across the sites. However, there were clear sociodemographic differences that likely contributed to the varying risk structures. Furthermore, all injection and sexual behaviours measures were self‐reported and collected by an interviewer and thus estimates may be over‐ or under‐reported and susceptible to social desirability bias. Self‐administered questionnaires or audio computer‐assisted self‐interview were not feasible and likely inappropriate given the limited education and poor literacy rates of the study population 62, 63. Questionnaires were administered face‐to‐face by trained interviewers with extensive experience with non‐judgemental interviewing techniques, thus reducing the potential for social desirability bias.

## 5 Conclusions

HIV treatment of PWID, whether for the prevention of HIV transmission or to increase the health and wellbeing of PWID living with HIV, presents unique, region‐specific challenges. While notable regional differences in the risk structure exist among our cohort, PWID within Ukraine have a unique vulnerability for a continued HIV epidemic that will require urgent efforts to address HIV transmission and acquisition. The regional differences in the risk structure among this cohort highlight the need for treatment as prevention interventions that are sufficiently flexible to address the needs of distinct PWID populations they are serving.

## Competing interests

The authors declare that they have no competing interests.

## Authors’ contributions

KEL, IFH, WCM and the HPTN Study Team had overall responsibility for implementing the study, and conceived and designed the study, analysed the data and led the manuscript writing. KEL, IFH, BH, TVH, KD, HS, SR, VFG, SRM, MGH, EMP‐M, PR, SD, ZD, TK, OZ, SD, CL, DM, DNB, JS, SAS, SHE, W, DD, LE, LES, LM, NS, ELH, JPL, BDD, NVV, RS, WCM and the HPTN Study Team contributed to developing the study concept and design. BH, TVH, KD, HS, SR, VFG, EMP‐M, PR, SD, ZD, TK, OZ, SD, CVA, SHE, WC, DD, LE, LES, LM, ELH and RS contributed to data collection. KEL, IFH, BH, SAR, KRM, MGH and WCM assisted with data analysis and results interpretation. KEL, IFH, WCM and the HPTN Study Team contributed to drafting the manuscript. All authors reviewed the manuscript critically for intellectual content. All authors read and approved the final draft of the submitted manuscript.
